# A newly developed high-performance thin layer chromatographic method for determination of remdesivir, favipiravir and dexamethasone, in spiked human plasma: comparison with the published methods

**DOI:** 10.1186/s13065-024-01366-1

**Published:** 2025-01-07

**Authors:** Rehab M. Abdelfatah, Esraa H. Abdelmomen, Eglal A. Abdelaleem, Refaat H. Abdelmoety, Aml A. Emam

**Affiliations:** 1 Pharmaceutical Analytical Chemistry, Faculty of Pharmacy, Beni-Suef University, Beni-Suef, Egypt; 2 Pharmaceutical Chemistry Department, Faculty of Pharmacy, Nile Valley University, Faiyum, Egypt; 3 Pharmaceutical Analytical Chemistry, Faculty of Pharmacy, Nahda University (NUB), Beni-Suef, Egypt

**Keywords:** COVID-19 antivirals, Co-administered, Corticosteroids, Whiteness

## Abstract

Co-administration of COVID-19 RNA polymerase inhibitors, remdesivir and favipiravir, has synergistic benefits. Together they reduce viral load and inflammation more effectively than either drug used alone. Corticosteroids like dexamethasone are used alongside antivirals in multidrug combination regimens. A new HPTLC method was utilized to isolate and quantitatively determine the three medicines of the COVID-19 therapeutic protocol, remdesivir, favipiravir and dexamethasone, using the anticoagulant apixaban as an internal standard in human plasma. The mobile phase system used a solvent mixture of ethyl acetate, hexane, and acetic acid (9:1:0.3, by volume). At 254 nm, well-resolved spots with Rf values of 0.3 for remdesivir, 0.64 for dexamethasone, and 0.77 for favipiravir have been observed. To ensure compliance with FDA regulations, a validation study was conducted. Quantitation limits as low as 0.1 µg/band have been achieved with remdesivir and dexamethasone, and 0.2 µg/band with favipiravir, demonstrating excellent sensitivities. From 97.07% to 102.77%, the drugs were recovered from human plasma that had been artificially spiked. The whiteness of the method has been assessed using RGB 12 algorithm and a percentage of whiteness of 95.6% has been obtained.

**Supplementary Information:**

The online version contains supplementary material available at 10.1186/s13065-024-01366-1.

## Introduction

More than 5.6 million people have died worldwide from 2019 coronavirus disease (COVID-19) [1], affected by severe acute respiratory syndrome coronavirus 2 (SARS-CoV-2). Despite the recent approval of numerous vaccines, the deadly pandemic continued unabated. This may be because there aren’t any effective therapeutic options available, or because vaccines and genetic modification aren’t widely available [2]. COVID-19 anti-viral drugs have been used to control the disease [3, 4]. Repurposing antiviral drugs already on the market, like Remdesivir (REM) and Favipiravir (FVP), is a feasible and effective strategy [5–7]. Effective suppression of SARS-CoV-2 replication can be achieved by using REM and FVP together [8].

Drugs like Dexamethasone (DEX) and anticoagulants were added to coronavirus protocols around the world because of their proven ability to alleviate the debilitating symptoms brought on by COVID-19 [9].

**Fig. 1:**
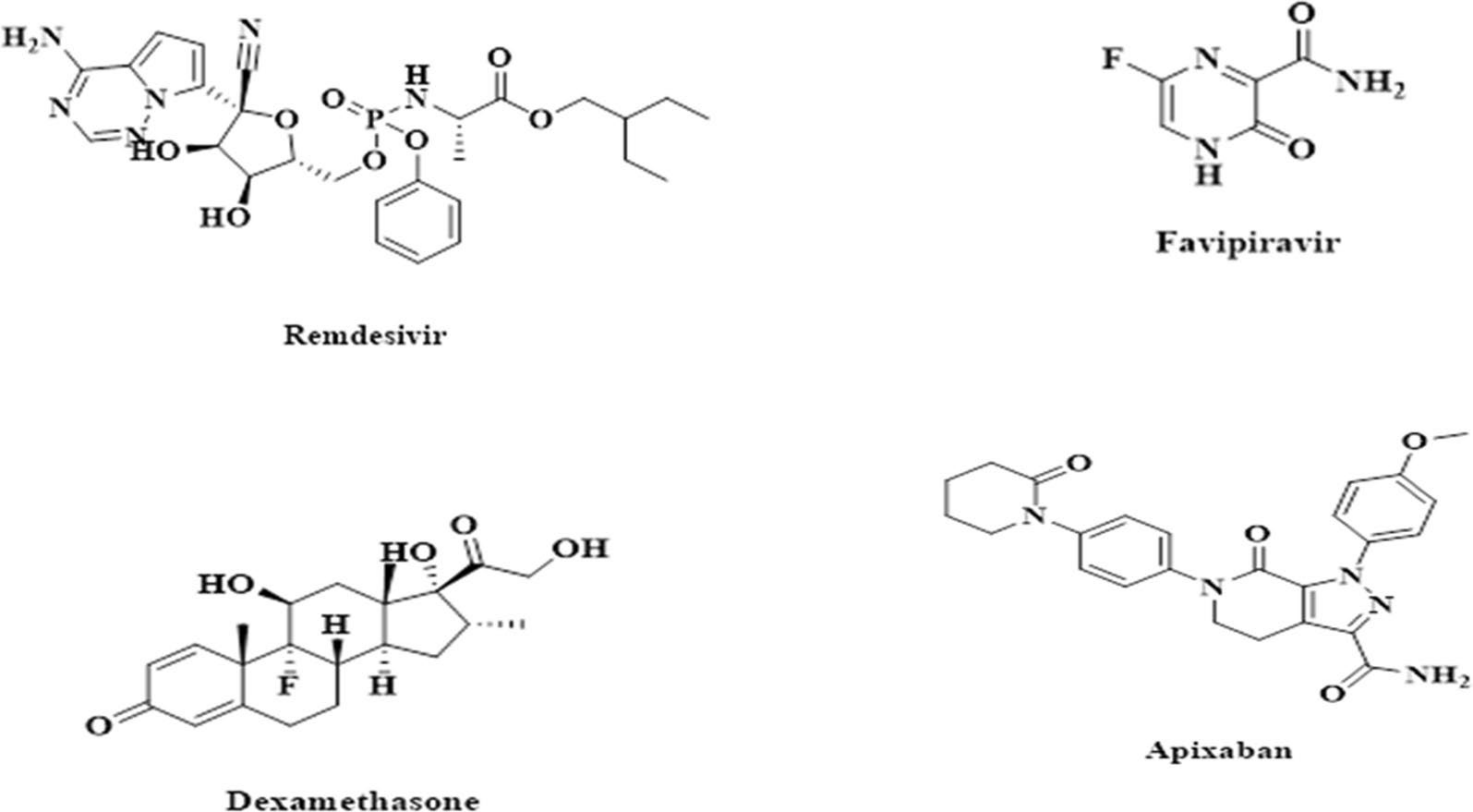
Chemical structure of Remdesivir (REM), Favipiravir (FVP), Dexamethasone (DEX) and Apixaban (PX)

REM is (2S) -2-[[[(2R,3S,4R,5R) ()-5-(4-aminopyrrolo [2,1-f] [1, 2, 4] triazin-7-yl) 5-cyano-3,4-dihydroxyoxolan-2-yl] methoxy-phenoxyphosphoryl] amino] Propanoate, Fig. 1, a compound first synthesized by Gilead Sciences for the treatment of Ebola virus infections [10]. Preliminary results suggested that REM aided recovery in hospitalized individuals with severe COVID-19. As the first medication of its kind, it received emergency approval from the US Food and Drug Administration (FDA) to treat hospitalized patients with COVID-19. Replication of coronaviruses can be stopped with antiviral drugs by blocking the enzyme RNA polymerase [11, 12].

Figure 1 shows 6-fluoro-3-hydroxy-2-pyrazine carboxamide (FVP), which was developed by Toyama Chemical Company in Japan as an influenza antiviral drug [13, 14]. Like REM, it blocks RNA polymerase enzymes and prevents viral replication. DEX, Fig. 1**,** is a glucocorticoid described for COVID-19 patients to reduce mortality [15, 16], and it has already been approved by the FDA for several uses.

To the best of our knowledge, REM was determined in human plasma by HPLC–MS/MS methods for therapeutic drug monitoring [17, 18], and in the presence of its major metabolite [19–21]. HPLC–DAD, spectrophotometric and spectrofluorimetric methods were used for its determination in dosage forms [22–25]. An electrochemical method was published for its determination in pure form [26]. FVP was determined in human plasma using HPLC–MS/MS methods [27–30] and an electrochemical one [31]. In pharmaceutical formulations, it was determined by HPLC–DAD [32–34], electrochemical [35] and spectrophotometric [34] methods. In the presence of its degradation products, it was determined by several methods including spectrophotometric, HPLC–DAD and HPLC–MS/MS [36–40]. DEX was determined in human plasma by HPLC–MS/MS [41, 42] and HPLC–DAD [43] methods. while it was determined in dosage forms by HPLC–DAD [44, 45] and spectrophotometric [46] ones. The previous methods have all been proposed for determining REM, FVP, and DEX independently. Few methods were described for the determination REM and FVP in human plasma without DEX including spectrophotometric [47], spectrofluorimetric [48, 49], TLC-Densitometric [50] and UPLC-MS/ MS [51]. One UPLC-DAD method was introduced for the determination of REM, FVP, and DEX [52].

Since many COVID-19 protocols call for concomitant administration of REM, DEX, and FVP [8, 9], we’ve created a sensitive and accurate HPTLC method for their simultaneous determination in human plasma to aid in adjusting therapeutic doses or applying it to pharmacokinetic studies. To compare our new method and the other published ones, white analytical chemistry criteria have been used. It was found that our new method is the best one on the practical side due to its low cost, time efficiency, low sample consumption and low practical requirements. Storing the medicines at -8 and -20 degrees Celsius, as shown by the freeze–thaw cycle, proved their stability. Advantages of the developed method include its sensitivity, durability, and selectivity which recommends its application to determine the three drugs in human plasma.

## Experimental

### Apparatus

Specifications of the apparatus and the equipment used for HPTLC chromatographic separation are listed in Table S1.

### Samples

Gifts of REM, DEX, and FVP with respective purities of 99.95%, 99.98%, and 99.87% were generously provided by Rameda Company (Cairo, Egypt).

Apixaban (PX), used as an internal standard, was kindly supplied by EVA Pharma (Giza, Egypt), and its purity was certified to be 98.28%. The National Egyptian Blood Bank generously donated blank plasma samples, which were stored at − 20 °C until needed. The samples were for six healthy volunteers 3 males and 3 females who received no medicine and their ages ranged from 18 to 45 years.

### Reagents and materials

The ethyl acetate and hexane used were of high purity grade (99.8% purity) and came from Riedel–dehaen, Sigma-Aldrich in Germany.

Acetic acid from EL NASR Pharmaceutical Chemicals Co., Abu-zabaal was of analytical grade (98% purity) from Cairo, Egypt.

### Standard solutions

Following an accurate weighting of 25 mg of REM, DEX, FVP, and PX, the powder was solubilized in 10 mL of methanol in each of the four 25 mL volumetric flasks. The volumes were filled with methanol to provide 1 mg/mL stock solutions for each component. 10 mL of the REM, DEX, FVP, and PX stock solutions were each placed into four 100 mL volumetric flasks to carry out further dilutions. The same solvent was then used to fill the volumes to the brim to create working solutions for every component.

### Chromatographic conditions

10 µL of each solution were spotted as 6 mm wide bands on TLC plates. Each band was 5 mm distant and spaced out 10 mm from the plate's bottom. The chromatographic tank was saturated for 30 min before the development was carried out to a depth of 9 cm with a solution of ethyl acetate, hexane, and acetic acid (9:1: 0.3, by volume). PX was applied as an internal standard. UV scanning at 254 nm was used to analyze the resulting bands.

### Analytical curves

Different REM, DEX, and FVP volumes were withdrawn from their respective 1 mg/mL stock solutions and placed in 10 mL volumetric flasks. Each flask was given an equal volume of PX, 1 mL of thawed plasma, and completed with methanol to make 0.1–10 µg/band of REM, 0.1–10 µg/band of DEX, 0.2–15 µg/band of FVP, and 5 µg/band of PX.

The solutions were prepared by stirring them in a vortex, centrifuging them at 4500 rpm for 10 min, and then filtering the supernatant through a syringe filter (0.45 µm Millipore).

## Results and discussion

This work created an HPTLC method for determining REM, DEX, and FVP that has been validated for its sensitivity, selectivity, speed, low cost, and low environmental impact. The developed HPTLC method offers the benefits of separating multiple analytes at once, using little solvent, and requiring little in the way of sample preparation. Resolution, Rf, peak sharpness and symmetry were optimized by changing the chromatographic parameters. The clinical dose of REM is 200 mg on day 1, followed by 100 mg for 12 days, resulting in a Cmax of 0.13–0.24 μg/mL, according to the age, within 0.68 h [53]. At the same time, the Cmax of DEX is 0.1 μg/mL within 2 h after a clinical dose of 6 mg daily [54]. The clinical dose of FVP is 1600 mg twice daily, reduced to 600 mg from the second day, the corresponding Cmax is 21.26 μg/mL within 0.5 h [55]. The proposed methods can quantitatively determine as low as 0.1, 0.1 and 0.2 µg/band of REM, DEX and FVP, respectively confirming its ability to estimate the serum concentrations of REM, DEX, and FVP in human plasma. Therefore, it can be used to monitor their therapeutic doses in COVID-19 patients.

### Method development and optimization

Multiple chromatographic parameters, including developing system composition, pH and detection wavelength were optimized to attain the most effective separation of REM, DEX, FVP, and PX.

#### Developing system selection

Various mixtures of green solvents like methanol, ethanol, and ethyl acetate were tested, beginning with ethanol: ethyl acetate (9:1, 7:3, and 6:4, v/v) and ethylacetate: ethanol (9:1, 7:3, and 6:4, v/v). When there was an incomplete separation between the three drugs and plasma. Also, FVP appeared near the front line. The addition of formic acid improves the separation of PX and DEX only. The addition of chloroform with ethyl acetate and formic acid to decrease the polarity of our developing system in the ratio (6:4:0.3, 7:3:0.3 and 5:5:0.3, by volume) gave good separation but FVP still on the front line. Replacing chloroform with hexane improved the separation to some extent.

#### pH optimization

pH plays a role in the proposed drugs' separation due to the presence of acidic and basic groups.

Formic acid, acetic acid, triethyl amine and ammonia solution (33%) were tested at volumes of 0.1, 0.2, 0.3, and 0.5 mL. The basic pH range was from 9.5 to 11.7 while the acidic range was 2.5 to 5. It was found that a pH of 4.5 provided the best separation. When comparing acetic acid and formic acid, acetic acid was found to be superior due to its ability to produce sharp and symmetric peaks.

#### Optimization of detection wavelengths

After trying scanning at 220, 240, 254, and 300 nm, we found that scanning at 254 nm provided the best and most sensitive results for all medications.

**Fig. 2:**
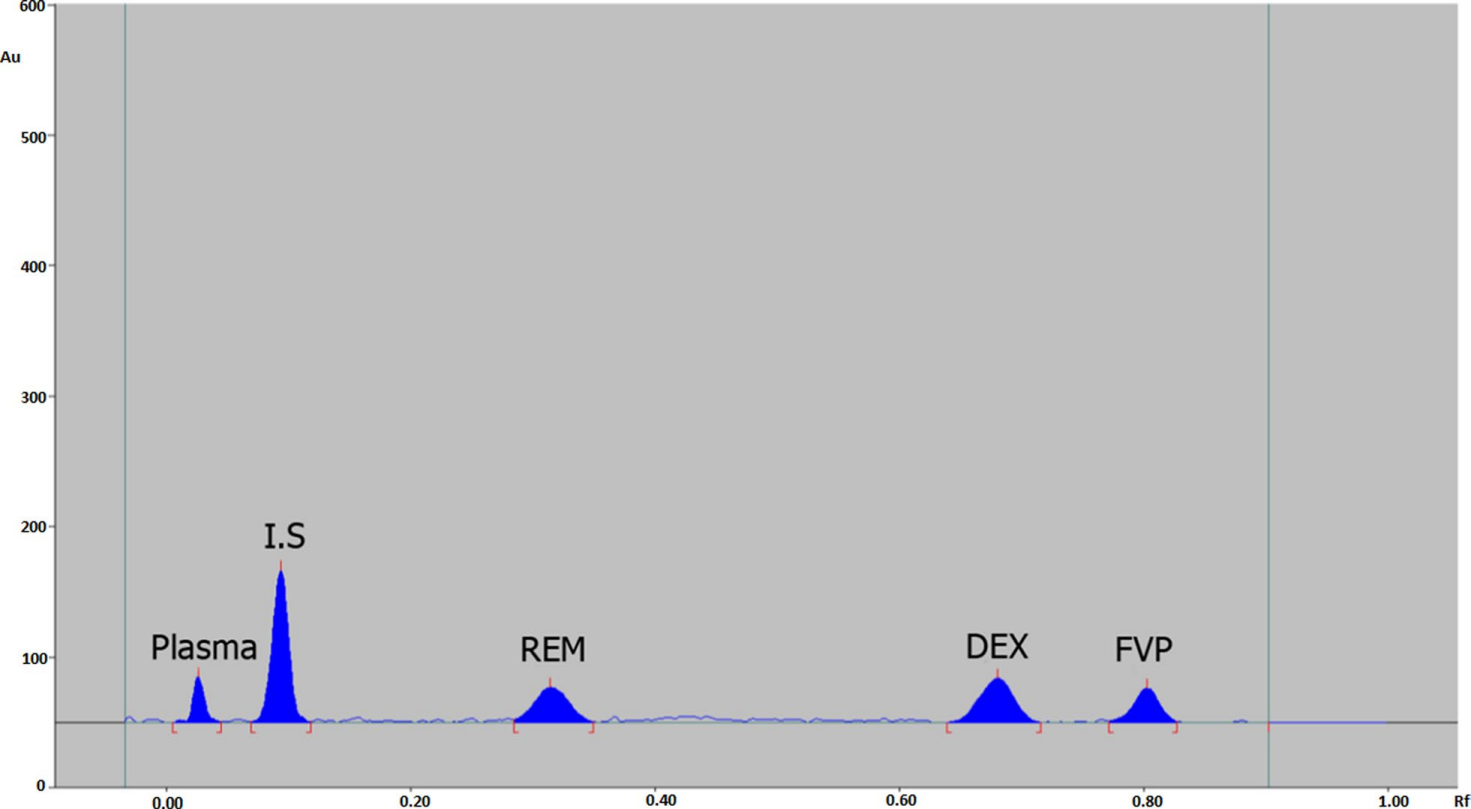
HPTLC chromatogram of human plasma spiked with PX (I.S), REM, DEX, and FVP using a developing system of ethyl acetate: hexane: acetic acid (9: 1.5: 0.3, by volume)

**Fig. 3:**
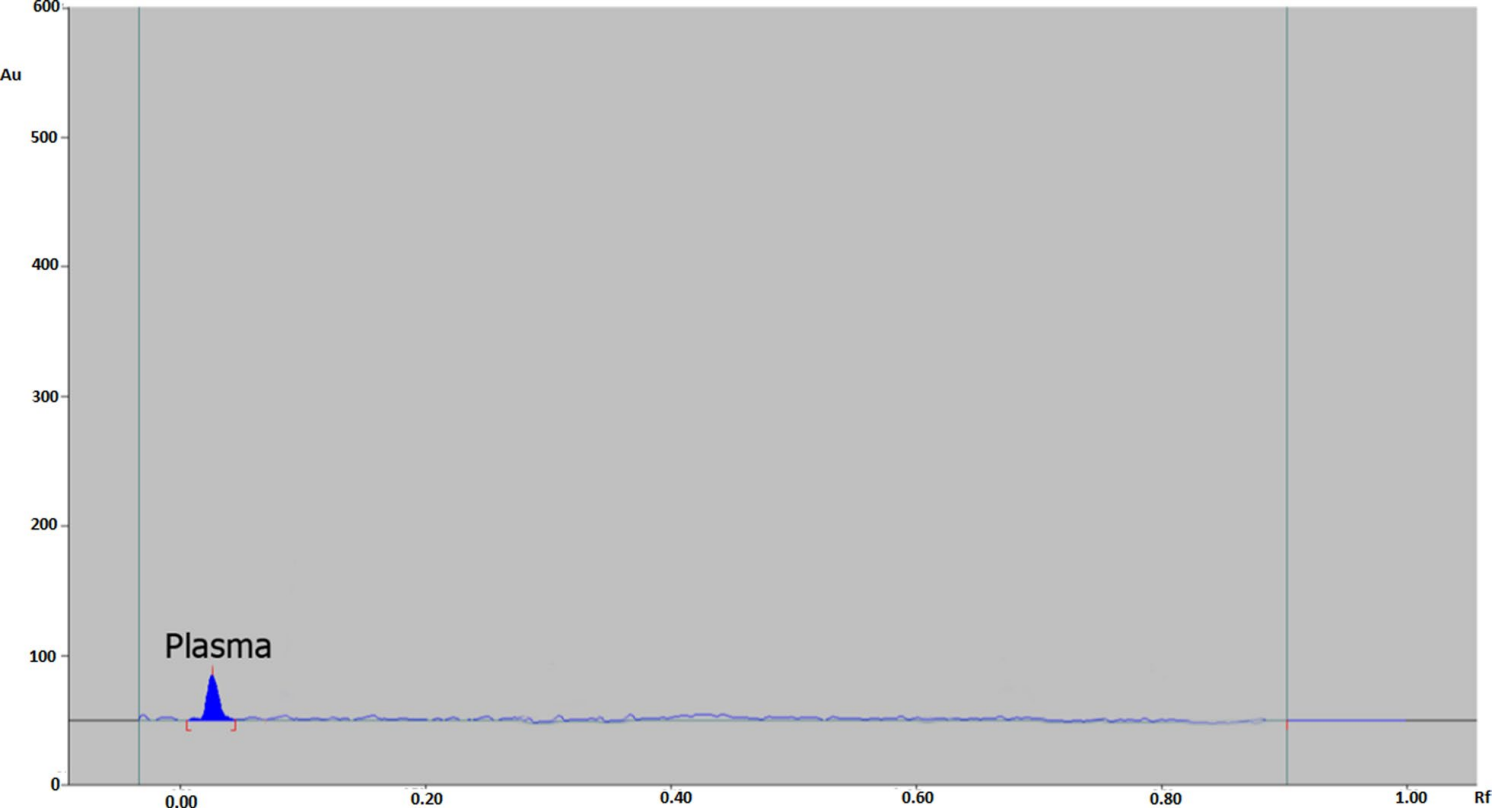
Chromatogram of blank human plasma

Finally, a mixture of ethyl acetate, hexane, and acetic acid (9:1:0.3, by volume) and scanning at 254 nm were found to be the optimal development conditions for REM, DEX, and FVP simultaneous measurement in plasma utilizing PX as an internal standard. 2D and 3D chromatograms of plasma spiked with the four drugs are displayed in Figs. 2 and 3, respectively. Plasma, PX, REM, DEX, and FVP were all found to have Rf values of 0.05, 0.1, 0.3, 0.64, and 0.77, respectively.

### Method validation

The recommendations of FDA Bioanalytical Method Validation Guidance for Industry were followed in the validation of the aforementioned procedure [56].

#### Range of linearity

Table 1 shows the calibrated plots for HPTLC peak area ratio calculations with 8 concentrations ranging from 0.1–10, 0.1–10, to 0.2–15 µg/band for the studied drugs The following regression equations were found:

**Table 1:** Analytical parameters for determination of REM, DEX and FVP by the proposed HPTLC method in spiked human plasma

Parameters	Spiked human plasma samples		
	REM	DEX	FVP
Calibration range (µg/band)	0.1–5 µg/band	0.1–5 µg/band	0.2–15 µg/band
Slope**	3.8000	3.2101	0.9371
Intercept	0.6153	1.3009	0.5403
Analytical curves			
Correlation coefficient	0.9999	0.9999	1
Accuracy (RSD %)*	96.32 ± 5.80	96.95 ± 5.01	99.01 ± 1.95
LLOQ (µg/band)	0.1	0.1	0.2
ULOQ (µg/band)	5	5	15

^*^Average of three determinations

^**^The linearity was achieved using the regression equation: A = aX^2^ + bX + C

a: coefficient 1. b: coefficient 2

A = peak area ratio (peak area of the analyte/peak area of IS) for spiked human plasma sample, X = concentration µg/ band. C = intercept

For REM,

$$ {\text{A1 }} = { 3}.{8}000{\text{ x }} + \, 0.{\text{6153 r }} = \, 0.{9999} $$

For DEX,

$$ {\text{A2 }} = { 3}.{21}0{\text{1 x }} - { 1}.{3}00{\text{9 r }} = \, 0.{9998} $$

For FVP,

$$ {\text{A3 }} = \, 0.{\text{9371 x }} + \, 0.{54}0{\text{3 r }} = \, 0.{9999} $$

At 254 nm, the peak area is denoted by A, concentration is denoted by x in μg/band, and r is the correlation coefficient. This confirms the developed method's linearity and its suitability for the estimation of the 3 drugs at their C _max_ values. Table 1 displays the regression and analytical parameters.

Additionally, for every drug, the lowest concentration was identified with a 20% precision (shown as % RSD) as listed in Table 1.

#### Accuracy and precision

Three replicates of each of the following concentrations were analyzed to determine the intra- and inter-daily precisions: (0.1, 0.4, 1, 4 µg/band) for REM and DEX; (0.2, 0.8, 3, 10 µg/band) for FVP. Table 2 demonstrates that respectable levels of accuracy were achieved (15% RSD).

**Table 2:** Intra and inter-day precision and accuracy of LLOQ, LQC, MQC and HQC of spiked plasma sample

Precision						
Drug	Concentration^a^ (µg/ band)^a^		Intra-day		Inter-day	
			Recovery %	RSD %	Recovery %	RSD %
REM	LLQC	0.1	99.13	1.21	95.56	0.89
	LQC	0.4	97.34	1.49	94.81	2.45
	MQC	1	100.16	1.71	96.92	2.88
	HQC	4	99.73	0.53	96.82	1.33
DEX	LLQC	0.1	101.07	2.16	100.43	2.74
	LQC	0.4	99.24	2.05	96.75	3.71
	MQC	1	99.22	2.73	97.84	2.87
	HQC	4	100.11	0.64	97.48	0.88
FVP	LLQC	0.2	98.08	0.99	98.08	0.99
	LQC	0.8	97.65	0.97	96.55	0.97
	MQC	3	97.18	1.97	98.53	0.30
	HQC	10	100.08	0.84	102.31	2.17

^a^Average of 3 experiments

#### Selectivity

Eight plasma samples underwent analysis using the developed method to identify plasma constituents that interfered with REM, DEX, FVP, and PX at their respective retention times. The plasma matrix does not affect REM, DEX, FVP, or PX as shown in Figs. 2 and 3.

#### System suitability parameters

System suitability parameters [57]including capacity factor, selectivity, symmetry factors, and resolution were calculated and compared to the reported chromatographic methods [50, 52]. Table 3 summarizes the results, which were satisfactory.

**Table 3:** Parameters of system suitability of the developed HPTLC for the determination of the proposed drugs and comparison with the reported methods

Parameters	Proposed method				Reported UPLC method [52]				Reported HPTLC method [50]		Reference (Srivastava, 2011) [57]
	PX	REM	DEX	FVP	PX	REM	DEX	FVP	FVP	REM	
Capacity factor (K′)	1.50	4.47	5.64	6.67	1.67	2.69	2.07	1.10	2.04	5.56	1–10
Symmetry factor	1.00	1.16	1.16	0.92	1.00	1.60	1.12	1.20	0.92	0.81	**~ 1**
Resolution (Rs)	4.91	4.91	6.86	2.50	1.73	–	2.50	2.74	6.38	–	Rs > 1.5
Selectivity (α)	2.98	2.98	1.27	1.18	1.24	–	1.30	1.52	10.73	–	α > 1

#### Extraction recovery

The recovery rates of REM, DEX, FVP, and PX from plasma were computed using the following formula, extraction recovery = (mean peak areas of the drugs in spiked plasma samples/mean peak areas of pure drugs in methanol). Four distinct concentrations were used to evaluate the extraction recovery (0.1, 0.4, 1, and 4 µg/band) for REM and DEX, and (0.2, 0.8, 3, and 10 µg/band) for FVP, Table 4.

**Table 4:** Extraction recovery results of the studied drugs in spiked human plasma by the proposed HPTLC method

Analyte	Concentration (µg /band)	Recovery % ± SD *
REM	0.1	88.51
	0.4	89.97
	1	90.03
	4	88.04
	Mean ± SD	89.14 ± 1.01
DEX	0.1	87.40
	0.4	87.55
	1	90.10
	4	88.90
	Mean ± SD	88.49 ± 1.27
FVP	0.2	84.88
	0.8	88.02
	3	87.92
	10	90.56
	Mean ± SD	87.84 ± 2.32

^*^Average of three determinations

#### Drug stability in biological fluid

The benchtop stability and freeze–thaw stability of REM, DEX, and FVP drugs in the plasma matrix were evaluated.

##### Bench top stability

At the beginning of the day, three concentrations of the frozen spiked plasma samples (low, medium, and high) were allowed to come to room temperature. Finally, the samples’ stability was assessed. The produced samples were stable during the analysis, as shown in Table 5.

**Table 5:** Stability results of the studied drugs in spiked human plasma at different conditions using the proposed HPTLC method

The analyte	Recovery %^a^		
	Concentration (µg/band)	Bench top stability	Three freeze thaw cycles^b^
REM	0.4	97.31	95.76
	1	100.18	99.24
	4	98.39	97.72
Mean ± RSD		98.63 ± 1.57	97.57 ± 1.78
DEX	0.4	98.24	97.94
	1	98.60	98.49
	4	99.79	99.06
Mean ± RSD		98.88 ± 1.07	98.49 ± 0.57
FVP	0.8	99.51	97.77
	3	98.91	97.13
	10	100.08	98.62
Mean ± RSD		99.19 ± 1.14	97.84 ± 0

^a^Average of 3 determinations

^b^Freezing was done at − 20 ºC

##### Freeze–thaw stability

Using the same three concentrations, spiked plasma samples were frozen overnight and then allowed to thaw at room temperature. The freeze–thaw cycle was repeated three times before substantial alterations were detected. Table 5 reveals that sample concentrations did not vary significantly after three cycles.

### Greenness assessment

Green analysis is characterized by the lack of or restricted use of risky chemicals, waste reduction, and energy consumption reduction[58]. The methods’ greenness profiles were evaluated using the National Environmental Method Index (NEMI) [59] and the eco-scale score [60]. NEMI focuses on four main criteria related to the solvents including the usage of persistent, bio-accumulative, and toxic (PBT) chemicals, corrosive reagents which assess whether corrosive substances are involved, hazardous waste which evaluates the potential for generating regulated hazardous waste and safety indicators which consider health and safety information. If the method meets NEMI green criteria, it is represented with a green circle. The developing system was a mixture of ethyl acetate-hexane-acetic acid (9: 1: 0.3, by volume) of a pH of 4.5 which wasn’t considered corrosive. Hexane is used in a minor proportion. The method produced trash amounting to 50 g including TLC plates, solvents, pipette tips, and filter papers. The graph produced after applying the NEMI tool is placed in Table 6. Moreover, an analytical eco-scale was implemented by assigning penalty points to method parameters. High Penalty Points are given for using hazardous reagents, large amounts of waste, and high energy consumption. In contrast, low Penalty Points are given for safer, more sustainable practices, then the penalty points are subtracted from 100. As shown in Table 6, the methods’ score of over 75 indicates excellent greenness. These results demonstrate that the proposed method is safe and environmentally friendly.

**Table 6:** Greenness assessment of the developed HPTLC method by NEMI and Analytical Eco-scale tools

Proposed HPTLC method	Analytical Eco-scale	NEMI
Reagent		
Ethly acetate	0	
Hexane	4	
Acetic acid	1	
Instrument TLC LC-UV < 1.5 kWh per sample	1	
Occupational hazards	3	
Waste (1–10 mL)	3	
Total penalty points	12	
Analytical eco scale	88	

### Comparison with the six reported methods regarding applicability

The new HPTLC method has been compared with the six reported ones [47–52] regarding the analyzed drugs, LOQ, the time required for analysis, internal standard and detection wavelength, Table 7. Our method has the advantage of separating 20 samples simultaneously in a single run, saving time compared to other methods. Also, it separates DEX along with REM and FVP. Although it isn’t the most sensitive one, it can determine the proposed drugs at their Cmax which is the main goal of this work. Also, a comparison regarding ANOVA and t-test was held between the methods regarding accuracy, Table S2. It can be concluded that no significant difference was found between them.

**Table 7:** Comparison between the created HPTLC method and the reported ones regarding performance and whiteness

	The proposed HPTLC method		UPLC-UV method [52]	Spectrophotometric method [47]			UPLC-MS [51]	Spectrofluorimetric method [49]			Spectrofluorimetric method [48]	TLC-Densitometric method [50]	
Investigated drugs	REM, DEX and FVP		REM, DEX and FVP	REM and FVP			REM and FVP	REM, FVP and hydroxyquinoline			REM and FVP	REM and FVP	
LOQ	0.1 µg/band for REM and DEX and 0.2 µg/ band for FVP		0.1 µg/mL for the three drugs	2 µg/mL for the two drugs			0.002 µg/mL for REM and 0.5 µg/mL for FVP	0.1 µg/for FVP , 0.05 µg/ mL for REM and 0.2 µg/ mL for hydroxyquinoline			0.02 µg/mL For REM and 0.04 µg/mL for FVP	0.12 µg/mL For REM and 0.07 µg/mL for FVP	
Analysis time per 20 samples	15 min		100 min	About 20 min			80 min	About 20 min			About 20 min	Not mentioned	
Internal standard	Apixaban		Apixaban	none			Acyclovir	none			none	none	
Detection wavelength	254 nm		240 nm	222, 256 and 228 nm for FVP and 247, 271, 251.2 nm for REM			m/z 157.9 > 112.92 for FVP and 603.09 > 200.005 for REM	423 nm for FVP, 384 nm for REM and 394 nm for hyroxyquinoline			251 nm for REM and 335 nm for FVP	235 nm	
Accuracy and precision	Are within the accepted criteria of the FDA guidelines which are ± 15% of nominal concentrations; except ± 20% at LLOQ												
Number of pictograms	12	6			3	7			6	5			13
Energy consumption	< 1.5 kWh per sample	< 0.1 kWh per sample			< 0.1 kWh per sample	1.5 kWh per sample			< 0.1 kWh per sample	< 0.1 kWh per sample			< 1.5 kWh per sample
Waste	More than 50 g with no proper management												

### Comparison with the six reported methods using white analytical chemistry criteria

**Fig. 4:**
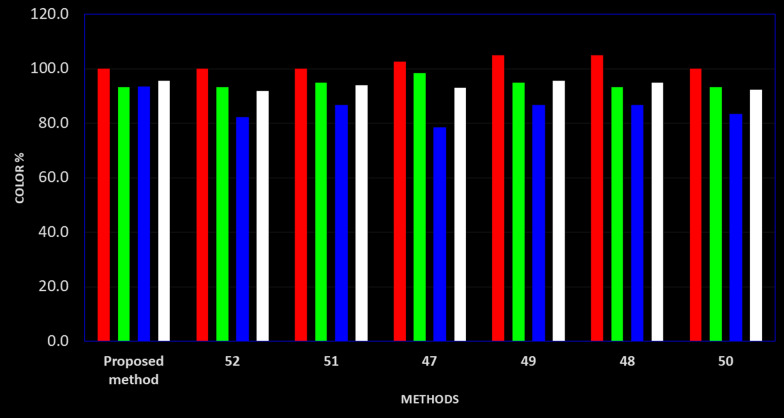
RGB 12 model for comparison between the proposed and reported methods

White analytical chemistry (WAC) [61] evaluates not only greenness but also the performance and practical applicability of a method. Moreover, it can compare up to 10 methods in the three aforementioned principles. WAC evaluates 12 parameters distributed equally between the three principles. RGB 12 model is used for the evaluation and comparison where R is for red principles evaluating method performance, G is for greenness principles and B is for practical side principles. Each of the evaluated principles is scored according to achieving the intended purpose. The combination of the scores of the three colors produces the whiteness of the method. The method performance depends on four parameters, the scope of application, LOD and LOQ values, accuracy and precision. Table 7 shows a comparison between the four parameters. The best scores were given to the most sensitive methods which are spectrofluorimetric ones [48, 49] followed by UPLC- Mass method [51] which has a very low LOQ value for REM concentrations. However, the LOQ values of our new method aren’t much greater than them. Concerning the second color, which is related to greenness, the number of pictograms, the amount of waste and energy consumption are compared as listed in Table 7. HPTLC, spectrophotometric and spectrofluorimetric methods are cost-effective if compared with UPLC-UV and UPLC-MS methods. Also, HPTLC methods are time effective as 20 samples can be determined simultaneously in a single run. This resulted in a high blue score for our method. Finally, the best whiteness score was for our proposed method and the spectrofluorimetric ones, but our method outperforms them in that it can analyze DEX simultaneously with REM and FVP which isn’t available in the spectrofluorimetric ones. Figure 4 represents the whiteness graph using the RGB model.

## Conclusion

To mitigate the spread of the coronavirus disease We developed a green, simple, and efficient HPTLC method as a first step toward applying it to in vivo studies, including pharmacokinetics and therapeutic drug monitoring, on the COVID-19 medications REM, DEX, FVP, and PX. The created method's eco-friendliness was also measured using the Eco-scale and NEMI tools while the whiteness of the method compared to other reported ones was evaluated by RGB 12 model. Results demonstrate that the proposed method is valid for application on human plasma according to FDA guidelines including linearity range, accuracy, precision, and stability, and is considerably safe for the environment, green, cheap cost, and time effective.

## Supplementary Information

Additional file 1.

## Data Availability

The datasets used and/or analyzed during the current study are available from the corresponding author on reasonable request.
