# Supplementary material for: A newly developed high-performance thin layer chromatographic method for determination of remdesivir, favipiravir and dexamethasone, in spiked human plasma: comparison with the published methods
Source: BMC Chem. 2025 Jan 7;19(1):7. doi: 10.1186/s13065-024-01366-1 (PMC11705924; doi:10.1186/s13065-024-01366-1)
Supplement: Supplementary file 1 — Additional file 1. [file 13065_2024_1366_MOESM1_ESM.docx]

**A newly developed high-performance thin layer chromatographic method for determination of Remdesivir, Favipiravir and Dexamethasone, in spiked human plasma: Comparison with the published methods.**

Rehab M. Abdelfatah^a^, Esraa H. Abdelmomen^b^, Eglal A. Abdelaleem^a^, Refaat H. Abdelmoety^c^ and Aml A. Emam^a^

^a^ Pharmaceutical Analytical Chemistry, Faculty of Pharmacy, Beni-Suef University, Beni-Suef, Egypt.

^b^ Pharmaceutical Chemistry Department, Faculty of Pharmacy, Nile Valley University, Faiyum, Egypt

^c^ Pharmaceutical Analytical Chemistry, Faculty of Pharmacy, Nahda University (NUB), Beni-Suef, Egypt

Corresponding author

Ass. Prof. Dr. Rehab M. Abdelfatah

Faculty of Pharmacy, Beni-Suef University, Beni-Suef, Egypt.

E-mail address: [r_magdy26@yahoo.com](mailto:r_magdy26@yahoo.com)

**Table S1: Specifications of the apparatus and equipment used for HPTLC chromatographic separation**

| **Apparatus and equipment** | **Manufacturer** |
| --- | --- |
| Camag TLC Scanner with winCATS software, (30.455 mm) slit dimension and (20 mm/s) scanning speed. | Muttenz, Switzerland |
| Camag Lino-mat 5 autosampler | Muttenz, Switzerland |
| TLC tank | Sigma-Aldrich® Co., USA |
| 20 x 20 cm silica gel aluminum plates (60 F254, 0.1 mm thickness) | Machenary-Nagel, Germany |
| Digital analytical balance AG 29 | Meltter Toledo, Glattbrugg, Switzerland |
| Tabletop low-speed centrifuge | TD3, Taiwan |
| Sonicator | Sonix TV ss-series, New York, USA. |

**Table S2**. Comparison between the created HPTLC method and the reported ones regarding accuracy using ANOVA and t-tests.

|  | | **The proposed HPTLC method** | **UPLC-UV method**  **[52]** | **Spectrophotometric method**  **[47]** | **UPLC-MS**  **[51]** | **Spectrofluorimetric method**  **[49]** | **Spectrofluorimetric method**  **[48]** | **TLC-Densitometric method**  **[50]** |
| --- | --- | --- | --- | --- | --- | --- | --- | --- |
| **ANOVA** | **REM** | The *f*-ratio value is 0.85455. The *p*-value is 0.50448. The result is *not* significant at *p* < 0.05. | | | | | | |
|  | **FVP** | The *f*-ratio value is 1.45328. The *p*-value is 0.253539. The result is *not* significant at *p* < 0.05. | | | | | | |
| **t-test**  **(2.228)*** | **REM** | - | 1.438 | 1.183 | Data isn’t available for comparison | 1.609 | 0.780 | 2.110 |
|  | **FVP** | - | 1.572 | 0.810 |  | 1.742 | 0.256 | 0.723 |

* The critical value of the t-test.
